# A ferroptosis associated gene signature for predicting prognosis and immune responses in patients with colorectal carcinoma

**DOI:** 10.3389/fgene.2022.971364

**Published:** 2022-09-08

**Authors:** Lijun Yan, Xi Chen, Zhaolian Bian, Chunyan Gu, Hanzhen Ji, Liyan Chen, Haifeng Xu, Qiyun Tang

**Affiliations:** ^1^ Department of Geriatric Gastroenterology, The First Affiliated Hospital with Nanjing Medical University, Institute of Neuroendocrine Tumor, Nanjing Medical University, Nanjing, China; ^2^ Department of Hepatology, Nantong Third People’s Hospital Affiliated to Nantong University, Nantong, China; ^3^ Department of Endocrinology, Taizhou Clinical Medical School of Nanjing Medical University (Taizhou People’s Hospital), Taizhou, China; ^4^ Department of Gastroenterology, Nantong Third People’s Hospital Affiliated to Nantong University, Nantong, China; ^5^ Department of Library, Nantong Third People’s Hospital Affiliated to Nantong University, Nantong, China

**Keywords:** colorectal carcinoma, ferroptosis, gene signature, prognosis, immune response

## Abstract

**Background:** Colorectal carcinoma (CRC) is one of the most prevalent malignancies globally. Ferroptosis, a novel type of cell death, is critical in the development and treatment of tumors.

**Objective:** This study was designed to establish a genetic signature for ferroptosis which has a predictive effect on the outcomes and immunotherapeutic response of CRC.

**Methods:** Data of CRC patients were retrieved from TCGA and GEO databases. The genes associated with ferroptosis were obtained from GeneCards. The genetic signature for ferroptosis was identified by performing Cox regression analysis. Kaplan–Meier and ROC analysis were performed to assess the prognosis role of the genetic signature. CIBERSORT tool was used to identify a potential association of the genetic signature with the immune cells. The potential immunotherapeutic signatures and drug sensitivity prediction targeting this signature were also discussed. Immunohistochemistry was used to detect expression of ferroptosis-associated genes in CRC tissues and adjacent tissues.

**Results:** A ferroptosis-associated gene signature comprised of three genes (CDKN2A, FDFT1, and ACSL6) was developed for prediction of prognosis and evaluation of immune responses in CRC. Patients in the high-risk group tended to have a poor prognosis. In CRC, the ferroptosis-associated gene signature may function as independent predictors. Additionally, the expressional levels of the immune checkpoint proteins PD-L1 and CTLA-4 were substantially increased in the high-risk group. Moreover, we can distinguish between patients based on their immunotherapeutic responses more effectively if we categorize them by this signature. Additionally, candidate compounds were identified for the differentiation of CRC subtypes.

**Conclusion:** The ferroptosis-associated gene signature identified in this study is effective in predicting the prognosis and evaluating immunotherapeutic response in CRC patients, and provides us with novel insights into the potential effect of ferroptosis targeted treatment on CRC.

## Introduction

Colorectal carcinoma (CRC) is one of common gastrointestinal system malignancies, ranking second in terms of carcinoma-related mortality worldwide (Siegel et al., 2020). The increasing disease burden caused by CRC has become one of the major public health problems. According to the GLOBOCAN Project 2018 of the WHO Cancer Research Centre, about 1.8 million new cases of CRC and about 880,000 deaths were reported in 2018 (Bray et al., 2018). It is beneficial for CRC patients to receive comprehensive treatments such as surgical removal, chemoradiotherapy, radiotherapy, immunotherapy and targeted therapy, but the present clinical treatment remains far from achieving ideal results, and CRC patients have a variation in individual prognosis (Wu, 2018; Ganesh et al., 2019; Zhang et al., 2019). Meanwhile, the 5-year survival rate in patients with CRC is about 64%, but decreases to 12% in those with metastatic CRC (Xie et al., 2020). Due to the heterogeneity of CRC patients, existing prognostic markers such as carcinoembryonic antigen (CEA) and tumor, lymph node, and metastasis (TNM) staging systems are inaccurate in predicting prognosis. Therefore, it is imperative to search for more precise biomarkers, and it is emergent to establish a better prognosis prediction model for CRC patients, which can help clinicians formulate appropriate treatment strategies.

Ferroptosis, a recently recognized nonapoptotic cell death, characterized by iron-dependent lipid peroxidation, differs from traditional types of cell death such as apoptosis, necroptosis, and autophagy (Dixon et al., 2012; Stockwell et al., 2017; Hirschhorn and Stockwell, 2019). Several studies have shown that ferroptosis is implicated with multiple diseases, such as tissue ischemia, neurodegenerative disorder, and tumor (Shi et al., 2019; Tang et al., 2022), which has garnered the attention of tremendous academicians worldwide. Moreover, it has been reported that ferroptosis exerts a great effect on gastric, pancreatic, hepatocellular, and colorectal carcinoma in the gastrointestinal system (Nie et al., 2018; Lorenzato et al., 2020). Emerging researches have shown that ferroptosis-targeted treatment can be used as a new promising alternative for current anticancer treatment, especially for treatment of malignancies resistant to traditional treatments (Hassannia et al., 2019; Liang et al., 2019; Chen et al., 2020). Additionally, some ferroptosis-associated genes including p53, DPP4, SLC7A11, and GPX4 were closely correlated with genesis, progression, and prognosis of CRC (Xie et al., 2017; Chen et al., 2020; Xia et al., 2020; Hong et al., 2021). Recently, several studies have mined public databases in order to explore prognostic signatures based on ferroptosis-associated genes in a variety of tumors, such as uveal melanoma, glioma, HCC, and pancreatic carcinoma (Du and Zhang, 2020; Tang B. et al., 2020; Tang R. et al., 2020; Luo and Ma, 2021; Zheng et al., 2021). However, few study has yet confirm whether ferroptosis-associated genes are correlated with the outcomes of CRC patients.

This study was designed to carry out a systematical evaluation on the prognostic values and multiple effects of the ferroptosis-associated genes in the immune responses of CRC. The mRNA expression profiles and related clinical data of CRC patients were collected from available datasets. Furthermore, according to the ferroptosis-associated genes in the TCGA cohort, a prognostic risk signature was identified and verified in the GEO cohort. Thereafter, a nomogram incorporating the risk signature and clinicopathological features was created to enhance the present prognostic evaluation of CRC patients. Subsequently, the potential connections of the prognostic genes with immune cells were assessed. Finally, the gene set enrichment analysis (GSEA) was conducted to explore the mechanism of action. In years to come, the ferroptosis risk signature and nomogram might help clinicians identifying prognosis and making individualized therapy decisions for CRC patients.

## Materials and methodology

### Datasets

The RNA-seq data and related clinical data of 453 CRC patients screened from The Cancer Genome Atlas (TCGA) (https://portal.gdc.cancer.gov/) were taken as a training set. Similarly, the survival information of 579 CRC patients collected from Gene Expression Omnibus (GEO) (https://www.ncbi.nlm.nih.gov/geo/) database (GSE39582) were taken as a validation set. The detailed clinical data was shown in Supplementary Table S1. A list of 105 ferroptosis-associated genes detailed in Supplementary Table S2 was obtained from GeneCards.

### Differentially expressed gene analysis

limma, a R software package was applied for detection of the differences in expressional levels of ferroptosis-associated genes between tumorous and adjacent non-tumorous tissues in the TCGA cohort. The filter criteria were set as follows: *p* value was less than 0.05 and logFC was more than 1. Heatmap and volcano plot were used to visualize differential genes. To explore the connections between the candidate prognostic ferroptosis-associated genes, a protein-protein interaction (PPI) network diagram was drawn by using STRING platform.

### Establishment and evaluation of a ferroptosis-associated genetic signature for prognosis prediction

The correlation between ferroptosis-associated genes and the overall survival (OS) in CRC patients was assessed by using univariate Cox regression analysis. The coefficients were then determined using multivariable Cox regression analysis. The formula for calculating the risk score of each patient was displayed as follows: risk score = $\sum_{\text{i}=1}^{\text{n}}\left(\text{coef mRNAi*expression of mRNAi}\right)$ , of which, coef indicated coefficient. Based on the median value of risk score, total patients in TCGA and GEO cohorts were divided into low-risk group and high-risk group respectively.

Moreover, Kaplan–Meier (K-M) analysis was conducted for comparing the difference in OS between both groups. In addition, a received operating characteristic (ROC) curve was drawn to evaluate the predictive effect of the risk signature in predicting the survival of CRC patients. Subsquently, univariate and multivariate Cox regression analyses were used to detect whether the risk score could be utilized as an independent risk factor for survival prediction in CRC patients. A prognosis nomogram integrating age, stage and risk score was constructed to assess the prognoses of CRC at 1-, 3-, and 5-years.

### Gene set enrichment analysis

GSEA was carried out to determine if the gene sets were obviously different between two groups. For each analysis, gene set permutation was performed for 1,000 times. The risk score was computed by using phenotype label. Important gene sets were classified as those with a normalized enrichment score (NES) more than one and a minimal *p*-value less than 0.05.

### Assessment of fractions of different immune cell subtypes, and estimation of immune and stromal content

CIBERSORT (https://cibersort.stanford.edu/), an analytical tool created by Newman et al. (2015), was utilized to determine the abundances of member cell types in a mixed cell population by using gene expression data. A normalized mRNA expression matrix and a CIBERSORT tool in CRC cohorts were used to detect the fractions of 22 subtypes of immune cells in two groups. Moreover, we used the Estimation of STromal and Immune cells in MAlignant Tumor tissues using Expression data (ESTIMATE) algorithm *via* the R package “estimate” to assess the degree of infiltration of tumor cells and various normal cells to determine the StromalScore, ImmuneScore, and EstimateScore (Yoshihara et al., 2013)(19).

### Exploration of the model in the immunotherapeutic treatment

We used the R package maftools to evaluate and summarize the mutation data. The tumor mutation burden (TMB) was estimated according to tumor-specific mutated genes (Wu et al., 2020). The TIDE algorithm was performed to predict the potential of the immunotherapeutic response (Xu et al., 2020).

### Exploration of potential compounds targeting ferroptosis-associated genetic signature in clinical treatment

To obtain potential compounds in the clinic for CRC treatment, we calculated the IC50 of compounds obtained from the GDSC website in the TCGA project of the CRC dataset. We used the R package pRRophetic to predict the IC50 of compounds obtained from the GDSC website in patients with CRC.

### The human protein atlas and immunohistochemistry

HPA is an open-access online database (http://www.proteinatlas.org/) that contains various images of protein expressions in cancerous and normal tissues (Uhlen et al., 2015). The immunohistochemical images of 3 ferroptosis-associated genes were retrieved from HPA database to confirm the results of bioinformatical analysis in this study.

To verify the results of this study, we collected 42 paired adjacent tissues and tumor tissues from our hospital to carry out immunohistochemistry. Immunohistochemistry was performed as described previously using (Liu et al., 2019; Zhu et al., 2021)anti- CDKN2A (ab270058, abcam) and anti- ACSL6 (ab229958, abcam) antibodies.

## Results

### Detection of different expression levels of ferroptosis-associated genes and assessment of their prognostic significance in the CRC TCGA cohort

**FIGURE 1:**
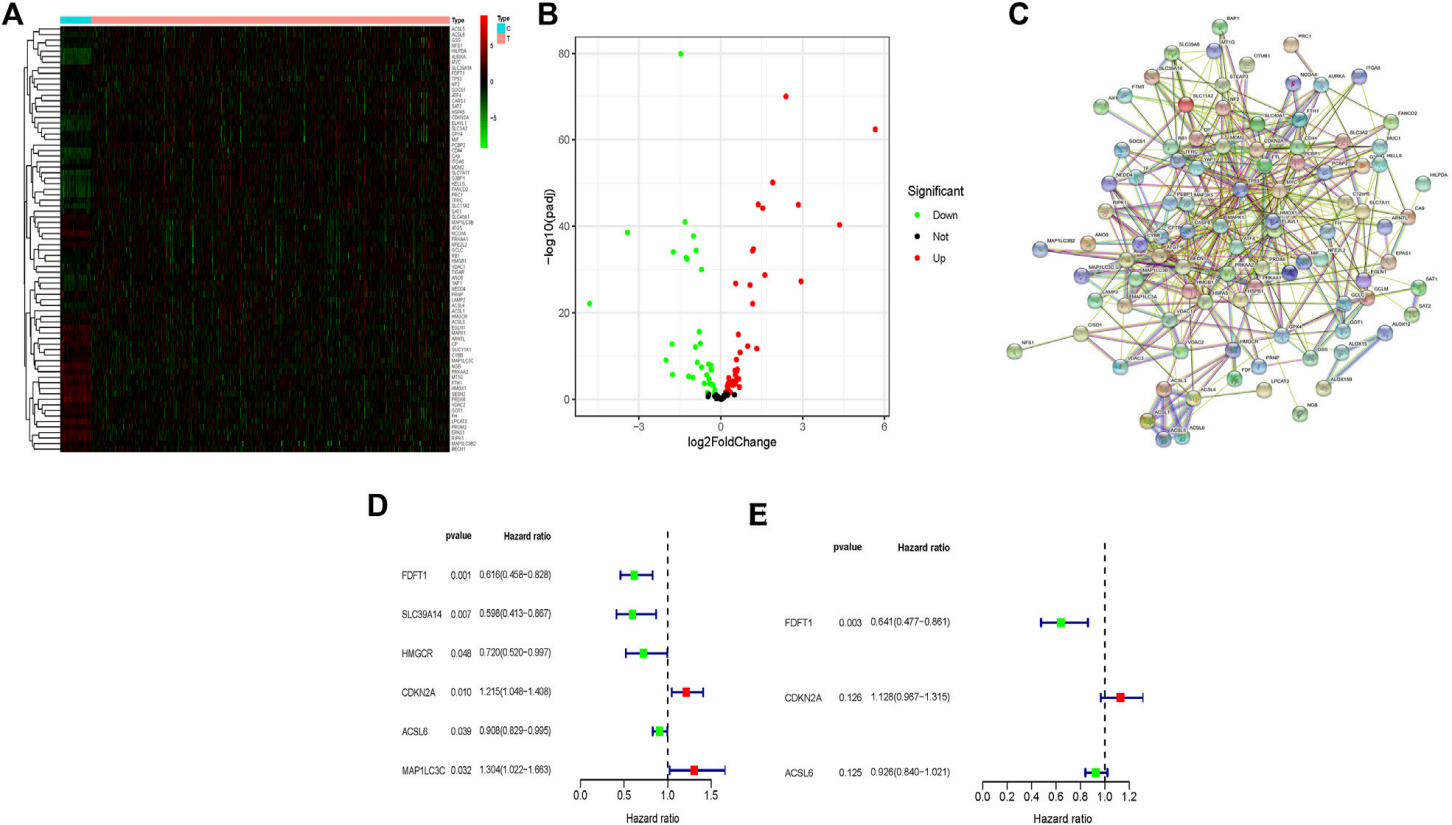
Identification of different expressional levels of ferroptosis-associated genes and their prognostic significance in CRC. **(A)** Different expressional levels of ferroptosis-associated genes in TCGA cohort were displayed in the heatmap and **(B)** the volcano map; **(C)** PPI network indicated the interactions among the candidate genes from the STRING; **(D,E)** a ferroptosis-associated gene signature was constructed by using univariate and multivariate Cox regression analyses to exert a predictive effect on the prognosis of CRC.

In this study, we used several advanced computational methods to systematically analyse the critical roles and prognosis effects of ferroptosis-associated genes in CRC. Flow chart of this study was illustrated in Supplementary Figure S1. The RNA-seq and clinical data of CRC patients were collected by retrieving TCGA and GEO databases. Figure 1A compared 77 ferroptosis-associated genes of different expression levels between the normal people (*n* = 41) and the CRC patients (*n* = 480) from TCGA, and they were also illustrated in the volcano map (Figure 1B), including 26 up-regulated genes and 51 down-regulated genes. STRING database (http://string-db.org) was retrieved to conduct protein-protein interaction network analysis, so as to achieve a better understanding of the interactions among above-mentioned ferroptosis-associated genes (Figure 1C). Moreover, the univariate Cox regression analysis was carried out to explore the prognosis significance of these ferroptosis-associated genes, and six prognostic-associated candidate genes were identified (Figure 1D), which implicated that CDKN2A and MAP1LC3C were prognostic risk genes for CRC patients. Meanwhile, FDFT1, SLC39A14, HMGCR and ACSL6 were genes protecting against CRC. Thereafter, multivariate Cox regression analysis was carried out to assess effects of these six prognosis-associated candidate genes on survival time and clinical outcomes of patients, and 3 ferroptosis-associated genes were determined as independent predictors in CRC (Figure 1E). Thereafter, the predictive model was constructed using 3 ferroptosis-associated genes. The risk score was computed using the formula as follows: risk score= (0.12 × CDKN2A)—(0.45 × FDFT1)—(0.08 × ACSL6).

### Prognosis effects of the three ferroptosis-associated gene signature in colorectal carcinoma patients

**FIGURE 2:**
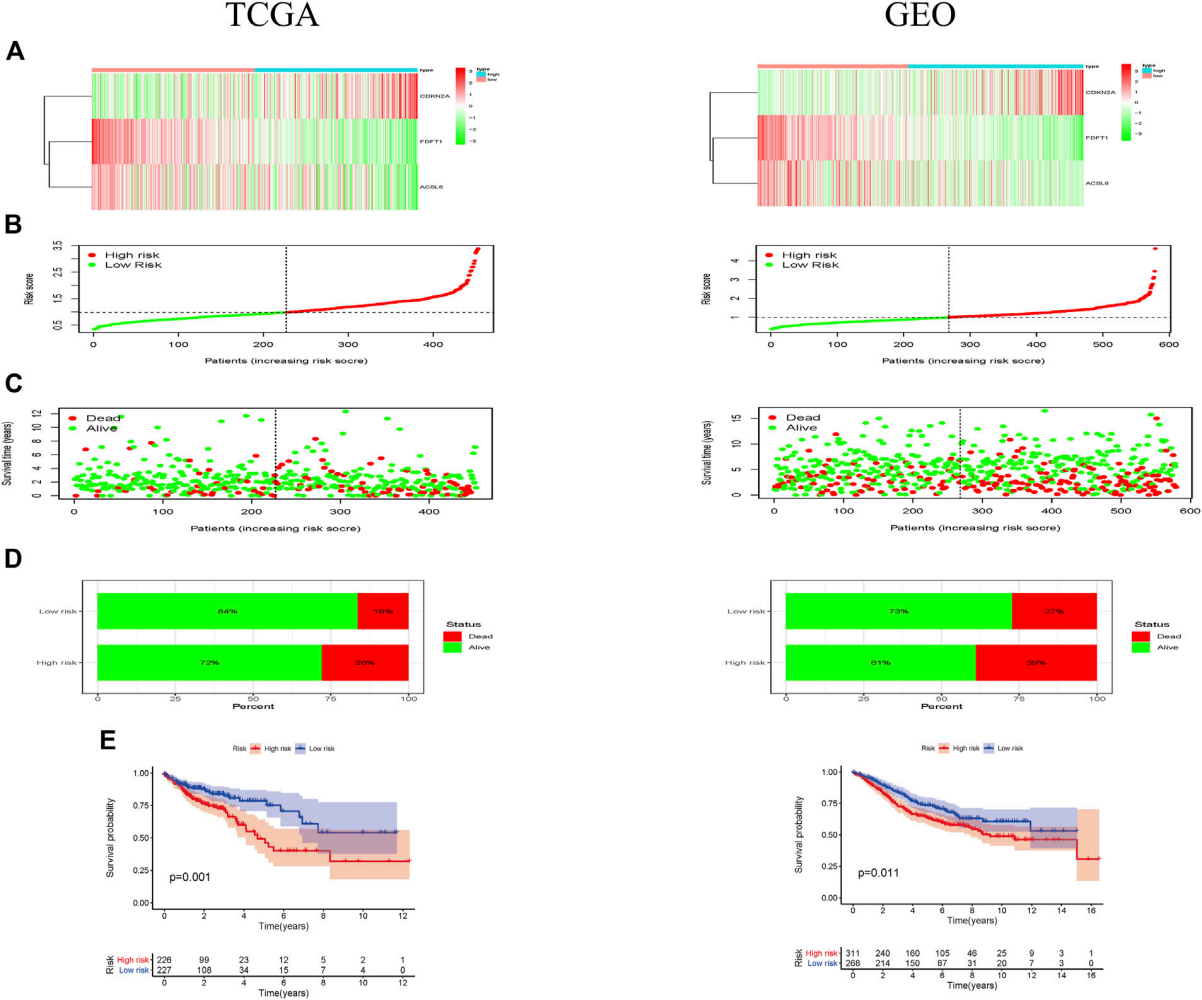
Prognostic effect of the ferroptosis-associated gene signature in CRC patients. **(A)** Heatmaps showed the expressional levels of 3 ferroptosis-associated genes respectively in low-and high-risk groups of TCGA and GEO cohorts; **(B)** the patients were grouped according to the ferroptosis-associated risk score. **(C)** The scatter plot demonstrated a difference in the survival status of CRC patients between low- and high-risk groups. The dot indicates the survival status of CRC patient, which ranked according to risk score in ascending order. **(D)** Mortality rates of the low- and high-risk groups; **(E)** Kaplan-Meier curves revealed a survival difference between two risk groups in TCGA and GEO cohorts.

As shown in the heatmap, 2 of 3 ferroptosis-associated genes had lower expression in the high-risk group both in TCGA and GEO cohorts (Figure 2A). Individuals in TCGA and GEO cohorts were classified into low-risk group and high-risk group by referring to their corresponding median risk scores (Figure 2B). Our data indicated that the patients had a higher mortality rate in high-risk group than that in low-risk group (Figures 2C,D). Furthermore, Kaplan-Meier analysis was performed for assessing the prognosis effect of the ferroptosis-associated genetic signature in CRC. As illustrated in Figure 2E, an increased ferroptosis risk score had a correlation with a worse overall survival (OS) in the TCGA cohort (*p* = 0.001), which was further confirmed in the GEO cohort (*p* = 0.011).

### Effectiveness of ferroptosis-associated gene signature in prognostic evaluation

**FIGURE 3:**
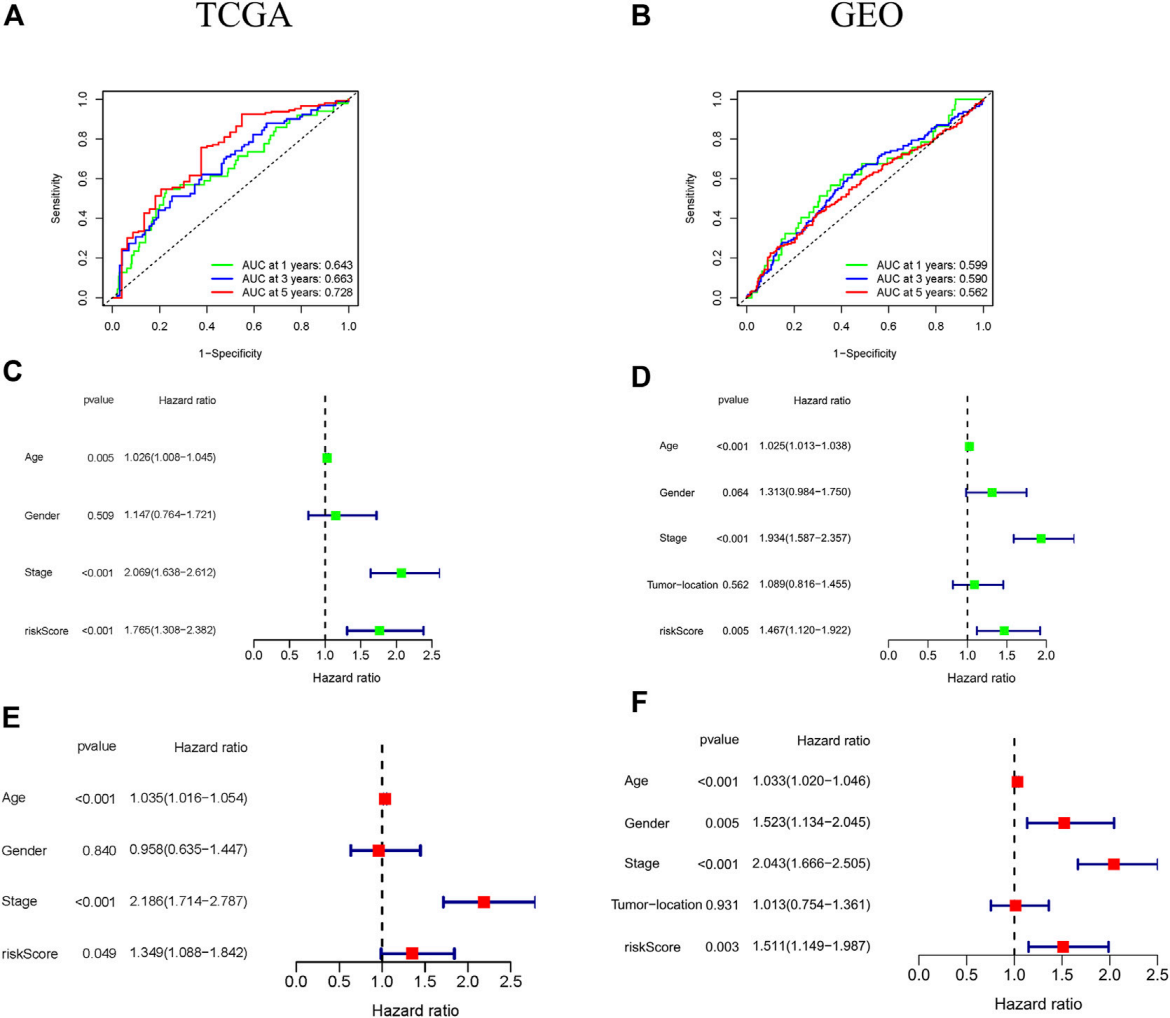
Prognostic significance of the ferroptosis-associated gene signature in CRC patients from the TCGA and GEO cohorts. **(A,B)** ROC curves indicated the accuracy of the ferroptosis-associated gene signature in the prediction of survival rates at 1-, 3-, and 5-years; **(C–F)** The independent prognostic significance of the ferroptosis-associated gene signature in OS in CRC patients using univariate and multivariate Cox analyses.

To assess the prognosis effect of ferroptosis-associated gene signature on 1-, 3-, and 5-year survival rates, the ROC curves were drawn by using the data respectively from TCGA and GEO cohorts. The area under the ROC curve (AUC) was 0.643 at 1-year, 0.663 at 3-years, and 0.728 at 5-years in TCGA cohort, suggesting that the ferroptosis-associated gene signature had a good predictive ability of the prognosis of CRC patients (Figure 3A). This was further confirmed in GEO cohort (Figure 3B).

The independent prognosis effect of ferroptosis-associated gene signature on OS of CRC patients was evaluated by performing univariate and multivariate cox regression analysis. The univariate cox regression analysis showed that age, stage, and risk score were independent prognostic predictors of OS (especially risk score, HR = 1.765, 95% CI = 1.308–2.382, *p* < 0.001) in TCGA cohort (Figure 3C). Furthermore, multivariate cox regression analysis indicated that age, stage and risk score had an independent association with significantly poorer OS in CRC patients (Figure 3E), suggesting that these variables could function as independent prognosis factors of CRC. These were confirmed in GEO cohort (Figures 3D,F).

**FIGURE 4:**
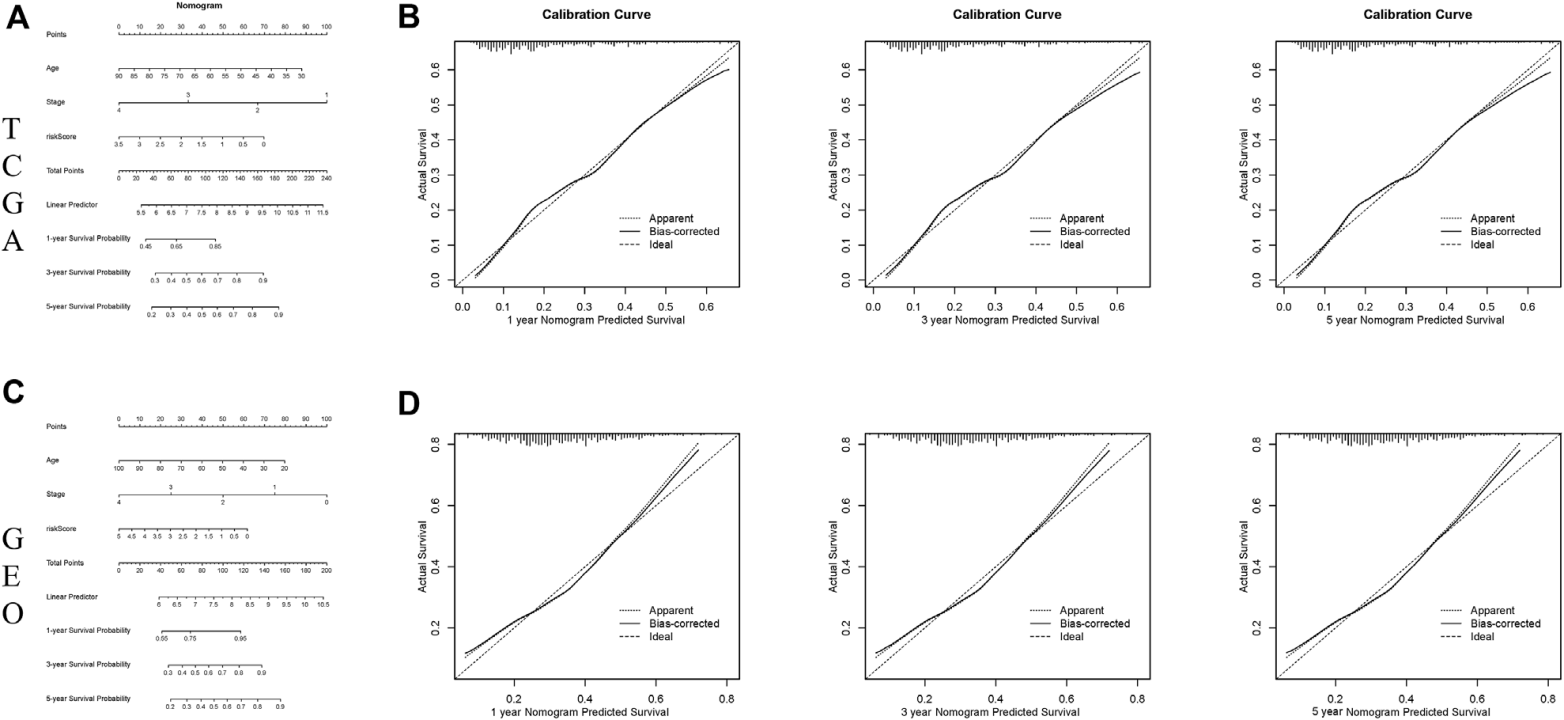
Constructing and verifying a nomogram. **(A,C)** The prognostic nomogram developed according to the risk scores of ferroptosis-associated genes and clinicopathological features predicted the 1‐, 3‐, and 5-year OS of CRC patients in the TCGA and GEO cohorts. **(B,D)** Calibration curves of nomogram on consistency between predicted and observed 1‐, 3‐, and 5-year survival in the TCGA and GEO cohorts.

The nomogram is a potent tool to quantify risk for patients in clinical environment through integration of many risk factors. Based on the above 3 variables (age, stage, and risk score), a prognostic nomogram was developed for predicting 1-, 3- and 5-year OS rates (Figures 4A,C). The calibration curves revealed that the actual survival rate was highly consistent with the predicted survival rate in both TCGA and GEO cohorts (Figures 4B,D), suggesting that this nomograph is accurate and dependable, and thus contributing to optimized clinical decision-making of clinicians for CRC patients.

### Relationship between ferroptosis-associated gene expression and clinicopathological features in colorectal carcinoma

**FIGURE 5:**
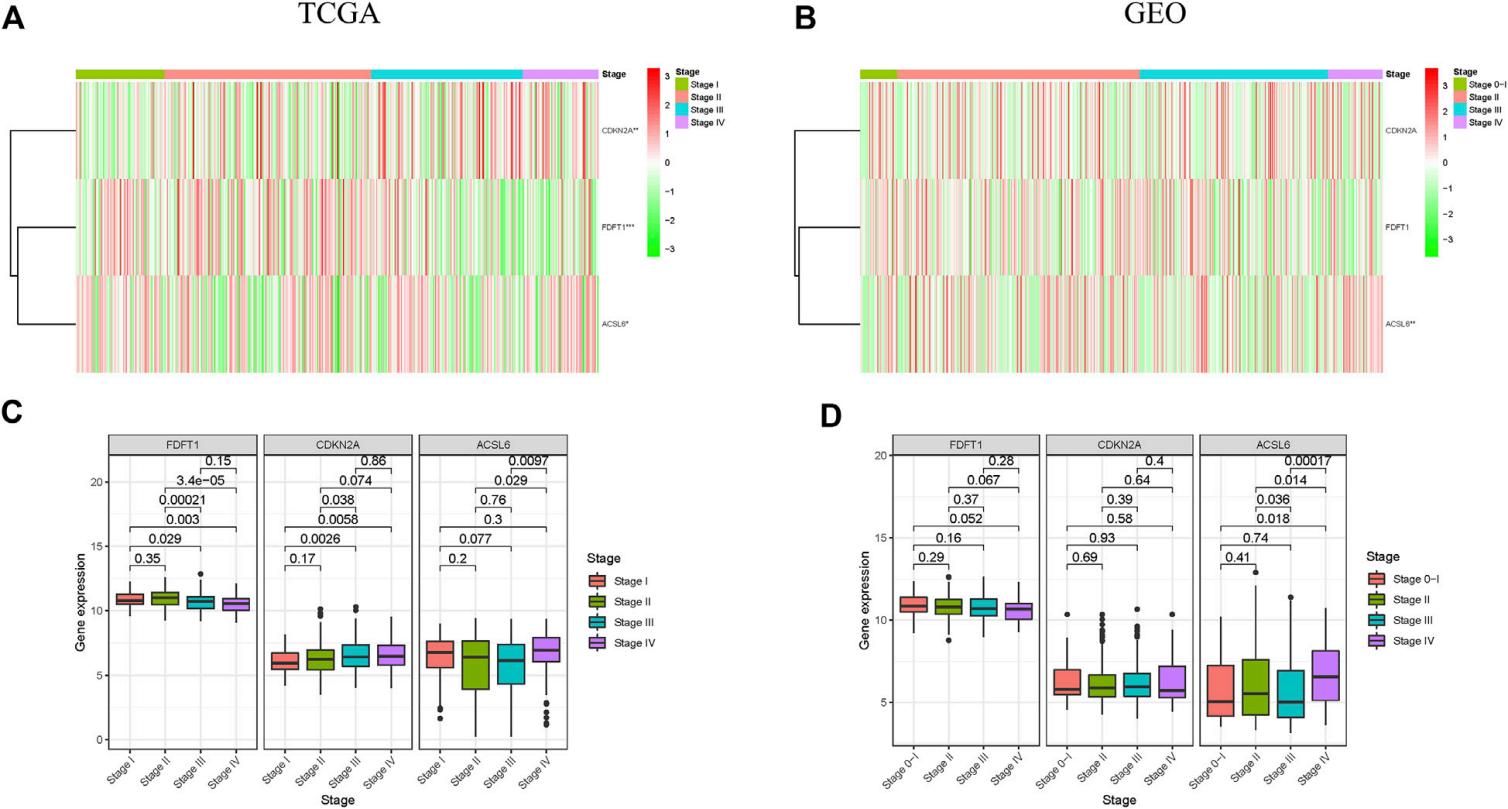
Correlation between ferroptosis-associated gene expressions and clinicopathological features in CRC patients. **(A,B)** Expression patterns of 3 ferroptosis-associated genes in different stages in TCGA and GEO cohorts; **(C,D)** Expression levels of 3 ferroptosis-associated genes in CRC at different stages in TCGA and GEO cohorts. *p < 0.05, **p < 0.01, and ***p < 0.001.

Additionally, the correlation of 3 ferroptosis-associated genes with clinicopathological features in CRC patients was investigated. Heatmap indicated the expression levels of 3 ferroptosis-associated genes at various clinicopathological stages in TCGA and GEO cohorts (Figures 5A,B). As drawn in Figure 5C, the expressional level of FDFT1, a presumed protective gene, was generally decreased in CRC patients with advanced clinicopathological stage in TCGA cohort. Conversely, the level of the risk gene CDKN2A was increased in CRC patients with advanced clinicopathological stage in TCGA cohort. The results of the CEO cohort were shown in Figure 5D.

### Relationship between ferroptosis-associated gene signature and immune cells

**FIGURE 6:**
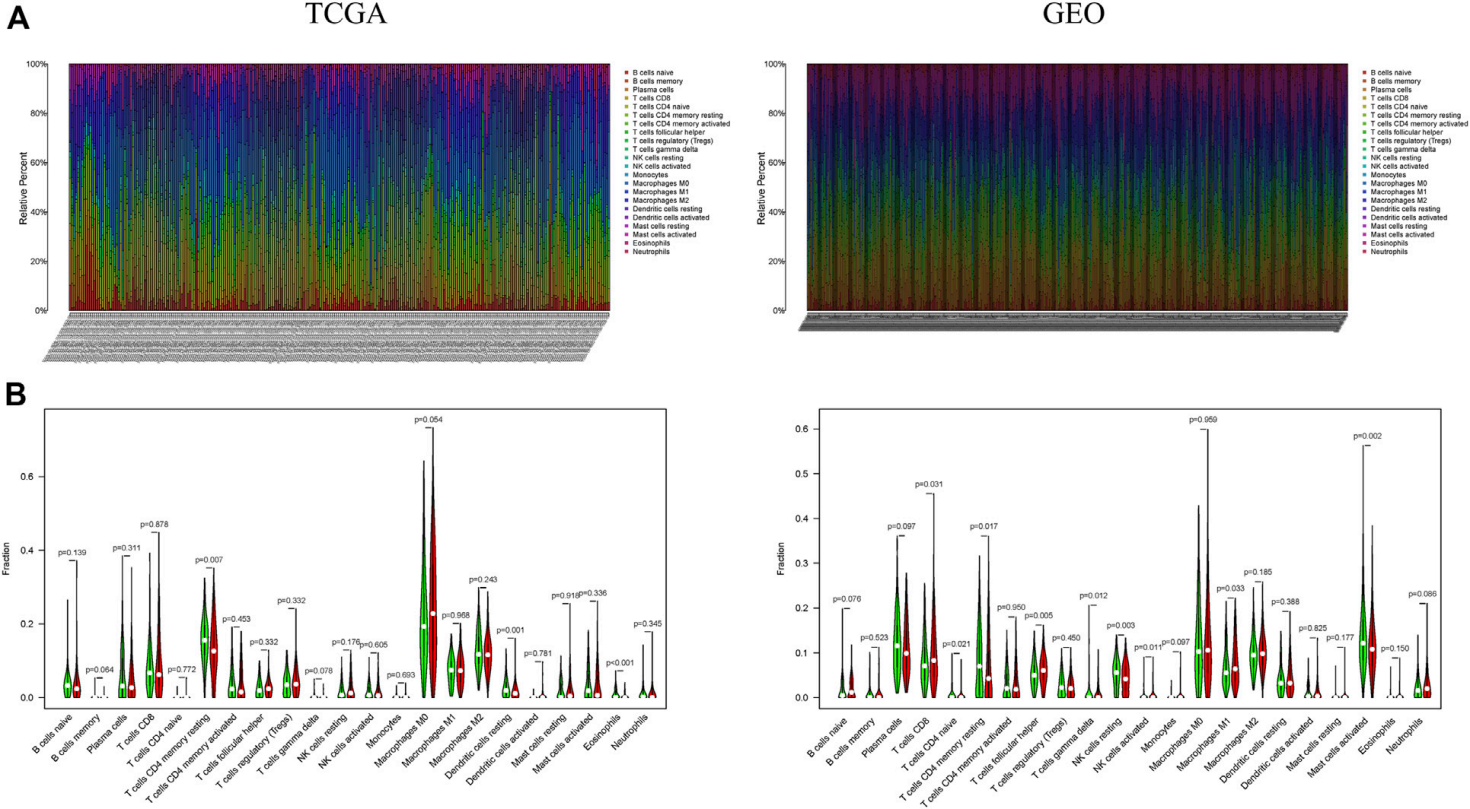
A difference in immune cell landscape between low and high ferroptosis-associated risks in CRC patients. **(A)** Relative distribution of 22 immune cells in all samples from TCGA and GEO cohorts; **(B)** The contents of immune cells in low- and high-risk groups. The low-risk group is indicated in green, the high-risk group is indicated in red.

The presence of 22 immune cell types were assessed in both TCGA and CEO cohorts (Figures 6A,B). In the TCGA cohort, there was an obvious difference in the presence of 3 types of immune cells (resting memory CD4^+^ T cells, resting dendritic cells and eosinophils) between 227 patients in the low-risk group and 226 patients in the high-risk group. In the GEO cohort, there was an obvious difference in the presence of 9 types of immune cells (CD8^+^ T cells, resting memory CD4 + T cells, naive CD4 + T cells, follicular T-helper cells, γδ T cells, activated natural killer (NK) cells, resting NK cells, M1-type macrophages, and activated mast cells) between 268 patients in the low-risk group and 311 patients in the high-risk group. In conclusion, there was an obvious difference in immune status between low- and high-risk groups, this needs to be further investigated to enhance cancer immunotherapy in CRC.

### Immunosuppressive microenvironment indicated by high ferroptosis-associated risk score

**FIGURE 7:**
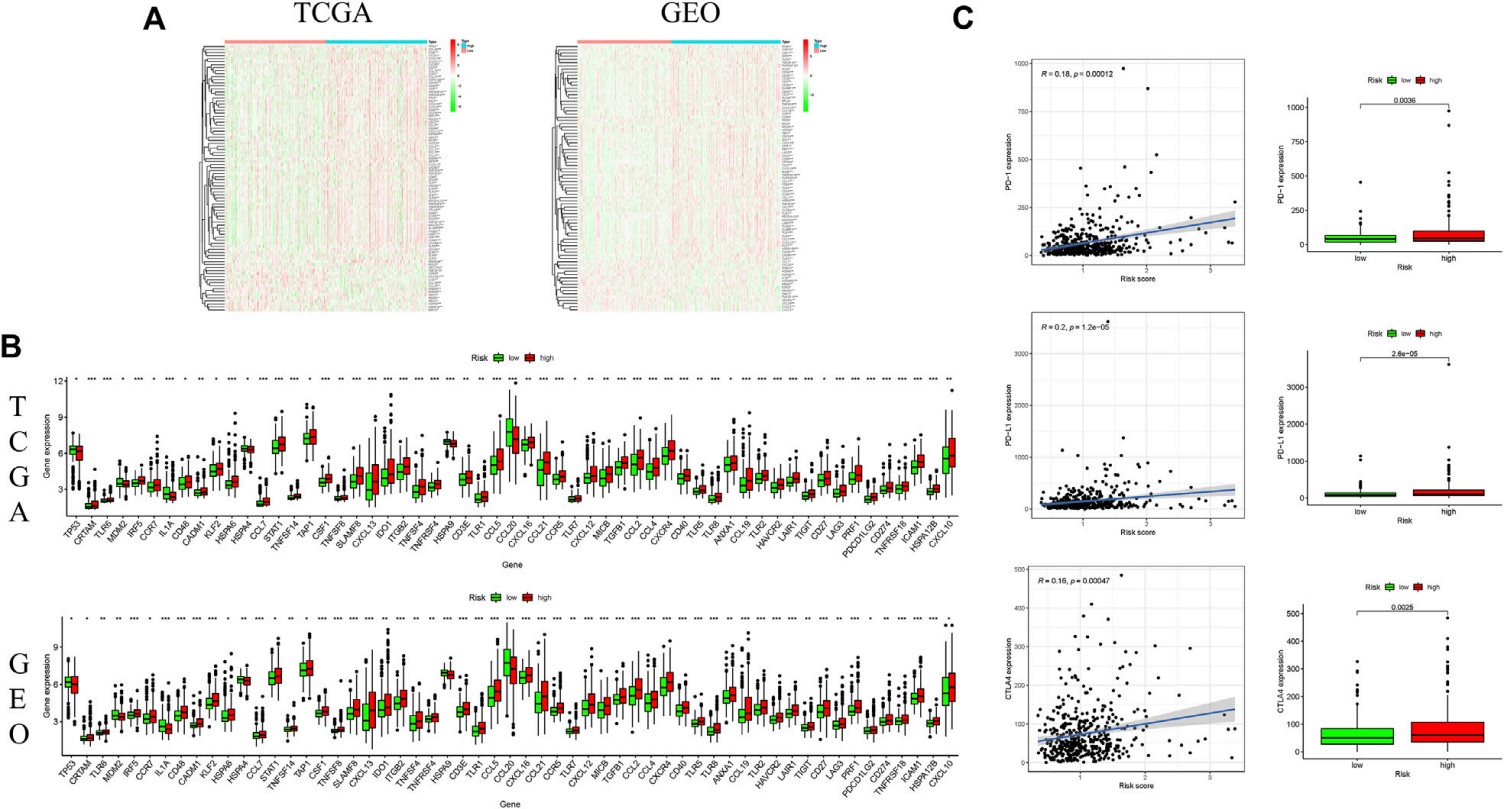
Correlation of ferroptosis-associated gene signature with immunity microenvironment. **(A)** Heatmaps of gene profiles of the cancer-immunity cycle in two risk groups in the TCGA and GEO cohorts; **(B)** comparison of the common differential immune gene expression between two risk groups in the TCGA and GEO cohorts; **(C)** comparison of immune checkpoint expression between two risk groups. *p < 0.05, **p < 0.01, and ***p < 0.001.

Genes signatures were derived from Tracking Tumor Immunophenotype website (http://biocc.hrbmu.edu.cn/TIP/). Heatmaps revealed that the genes negatively regulating the cancer-immunity cycle were obviously increased in the high-risk group, suggesting that patients in this group had decreased immunological competence (Figure 7A). The common differential genes were extracted in the TCGA and GEO cohorts, indicating that there was an obvious difference in expressions of these genes between two groups (Figure 7B).

In consideration of the important role of checkpoint inhibitor cancer immunotherapy, the difference in immune checkpoint expression was compared between low-and high-risk groups, and it was discovered that there was a substantial difference in the expressions of PD1, PDL-1 and CTLA4 between two groups (Figure 7C). These findings indicated that patients with increased ferroptosis-associated risk scores are prone to develop an immunosuppressive microenvironment due to increased immunosuppressive cytokines and immune checkpoints.

### Gene set enrichment analysis for identification of ferroptosis-associated signaling pathways

**FIGURE 8:**
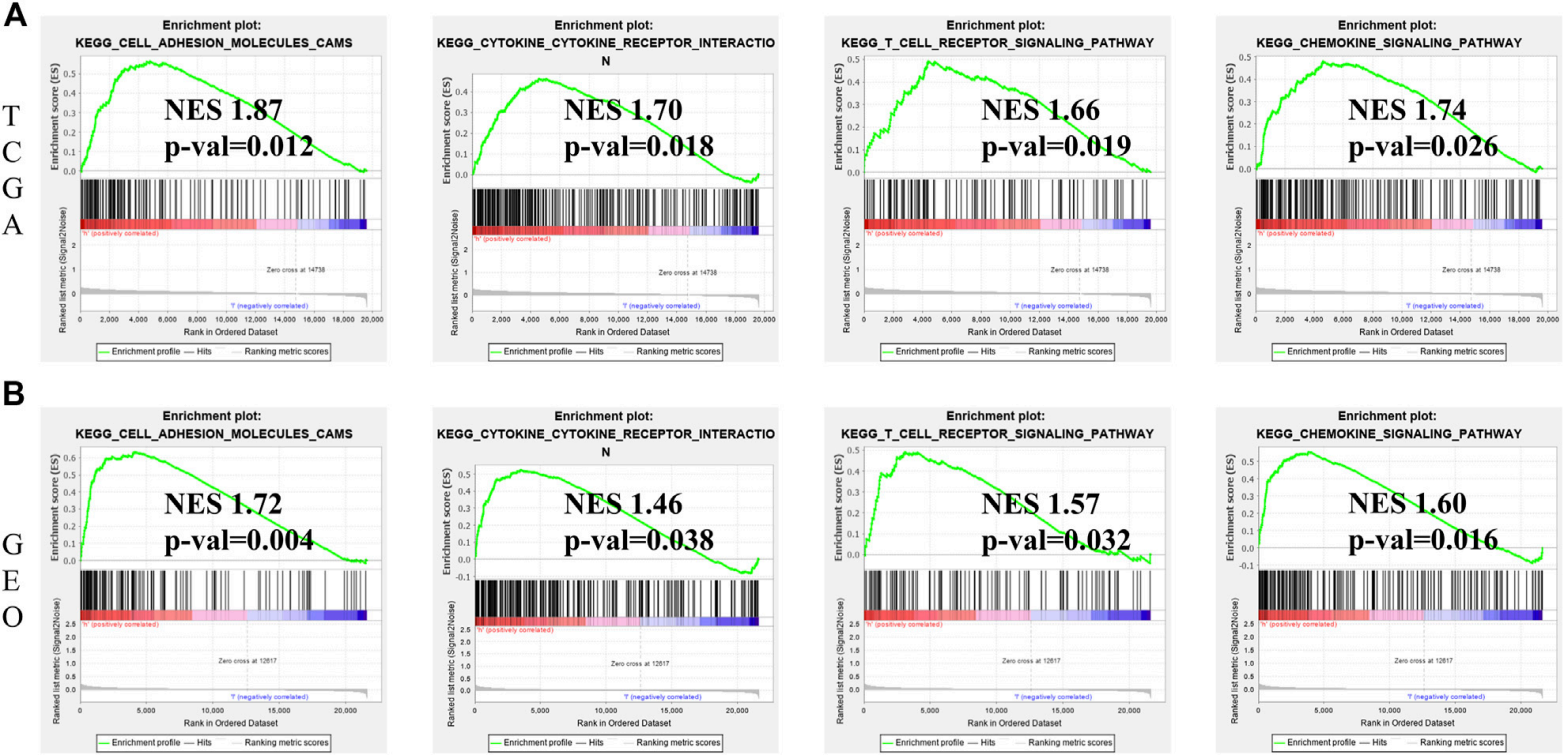
GSEA for identification of ferroptosis-associated signaling pathways. **(A)** GSEA of related signaling pathways in the high-risk group in TCGA cohort. **(B)** GSEA of related signaling pathways in the high-risk group in GEO cohort.

GSEA was performed to comparatively analyse the biological signaling pathway between low- and high-risk groups. Notably, in both TCGA and GEO cohorts, the gene sets associated with cytokine-cytokine receptor interaction, cell adhesion molecules, T cell receptor signaling pathway and chemokine signaling pathway were greatly enriched in the high-risk group (Figures 8A,B).

### Estimation of the tumor immune microenvironment and cancer immunotherapy response using the ferroptosis-associated gene signature

**FIGURE 9:**
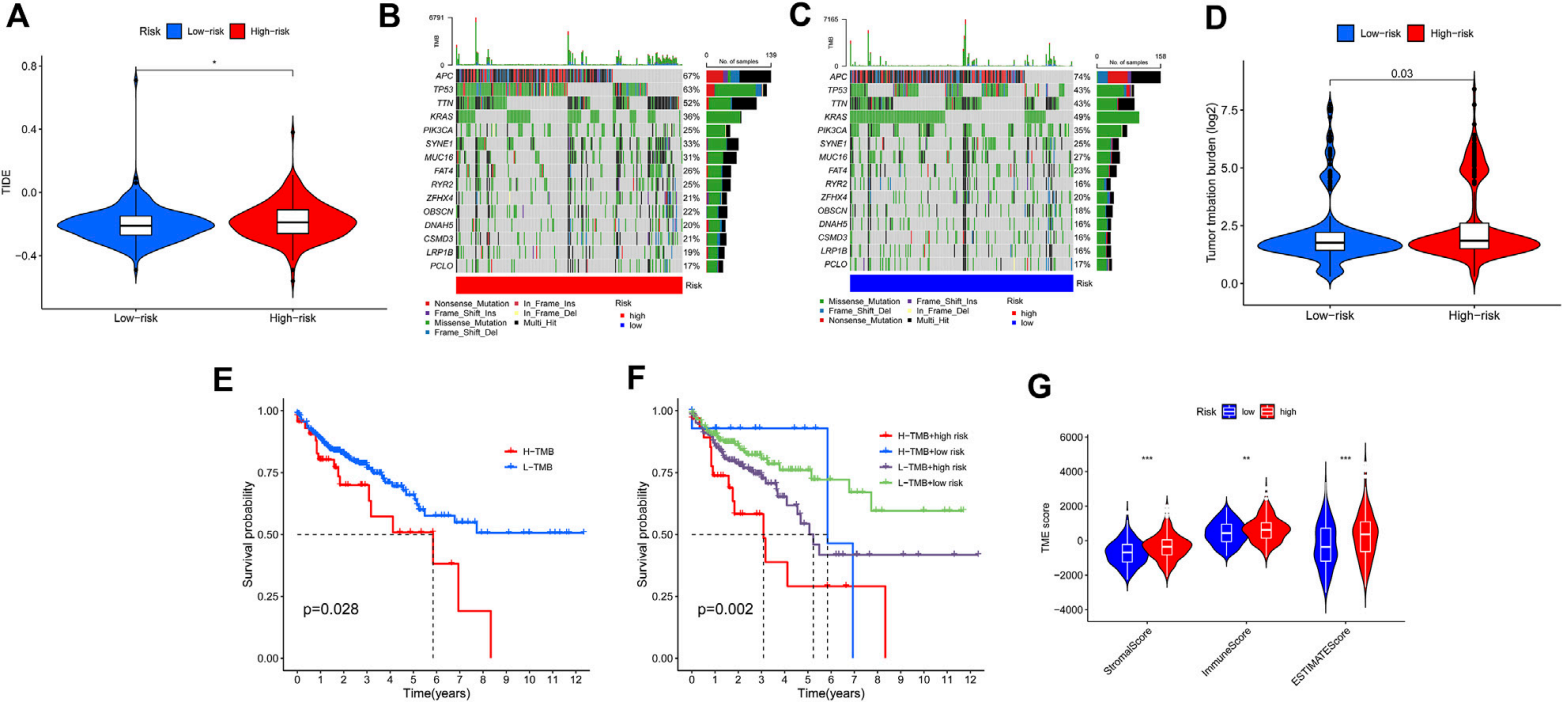
Estimation of the tumor immune microenvironment and cancer immunotherapy response using the ferroptosis-associated gene signature in the TCGA entire set. **(A)** TIDE prediction difference in the high- and low-risk patients. **(B,C)** Waterfall plot displays mutation information of the genes with high mutation frequencies in the high-risk group **(B)** and low-risk group **(C)**. **(D)** TMB difference in the high- and low-risk patients. **(E)** Kaplan-Meier curve analysis of OS is shown for patients classified according to the TMB status. **(F)** Kaplan-Meier curve analysis of OS is shown for patients classified according to the TMB status and ferroptosis-associated gene signature. **(G)** Comparison of Stromal_score, Immune_score and ESTIMATE_Score between two groups.

We next investigated the correlations between the ferroptosis-associated gene signature and immunotherapeutic biomarkers. Unsurprisingly, we discovered that the high-risk group was more likely to respond to immunotherapy than the low-risk group, indicating that this ferroptosis-based classifier index might serve as an indicator for predicting Tumor Immune Dysfunction and Exclusion (TIDE) (Figure 9A). Then, we used the R package maftools to analyse and sum the mutation data. Based on the variant effect predictor, the mutations were stratified. The top 15 driver genes with the highest alteration frequency between two groups are shown in Figures 9B,C. We then calculated TMB scores based on the TCGA somatic mutation data. The scores of TMB in the high-risk group exceeded that in the low-risk group (Figure 9D). Moreover, high score of TMB (H-TMB) is correlated with a worse survival and can be used as a prognostic marker in CRC (Figure 9E). Therefore, we tested whether the ferroptosis-associated gene signature could predict the OS outcome better than TMB scores. Patients with TMB in the high-risk groups (defined as “H-TMB + high risk” and “L-TMB + high risk”, respectively) presented a worse OS than patients with TMB in the low-risk groups (defined as “H-TMB + low risk” and “L-TMB + low risk”, respectively) (Figure 9F). Interestingly, patients with L-TMB in the high-risk group had worse survival outcomes than patients with H-TMB in the low-risk group. The survival curve of patients with H-TMB was similar to that of patients with L-TMB in the high-risk group, indicating that the TMB scores failed to distinguish the survival rate in the high-risk group. Thus, these findings indicate that the ferroptosis-associated gene signature may have greater prognostic significance than the TMB scores.

According to the results of ESTIMATE, the tumor microenvironment characteristics between two groups were identified. We found that the high-risk group had higher levels of StromalScore, ImmuneScore, and ESTIMATEScore, whereas the low-risk group had lower levels of these scores (Wilcox test, *p* < 0.01) (Figure 9G).

### Identification of novel candidate compounds targeting the ferroptosis-associated gene signature

**FIGURE 10:**
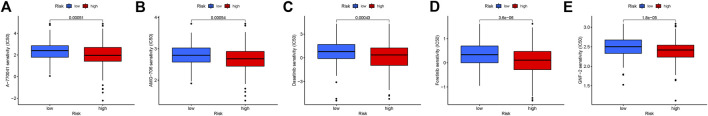
**(A–E)** Identification of novel candidate compounds targeting the ferroptosis-associated gene signature.

To identify potential drugs targeting our ferroptosis-associated gene signature for treating CRC patients, we used the pRRophetic algorithmto estimate the therapeutic response based on the half-maximal inhibitory concentration (IC50) available in the Genomics of Drug Sensitivity in Cancer (GDSC) database for each sample. We found that 12 compounds were screened out for significant differences in the estimated IC50 between these two groups, and the high group was more sensitive to all of these compounds. Figure 10 shows the top 5 compounds that might be used for further analysis in patients with CRC.

### Verification of the expressions of ferroptosis-associated genes in colorectal carcinoma and normal colorectal tissues

**FIGURE 11:**
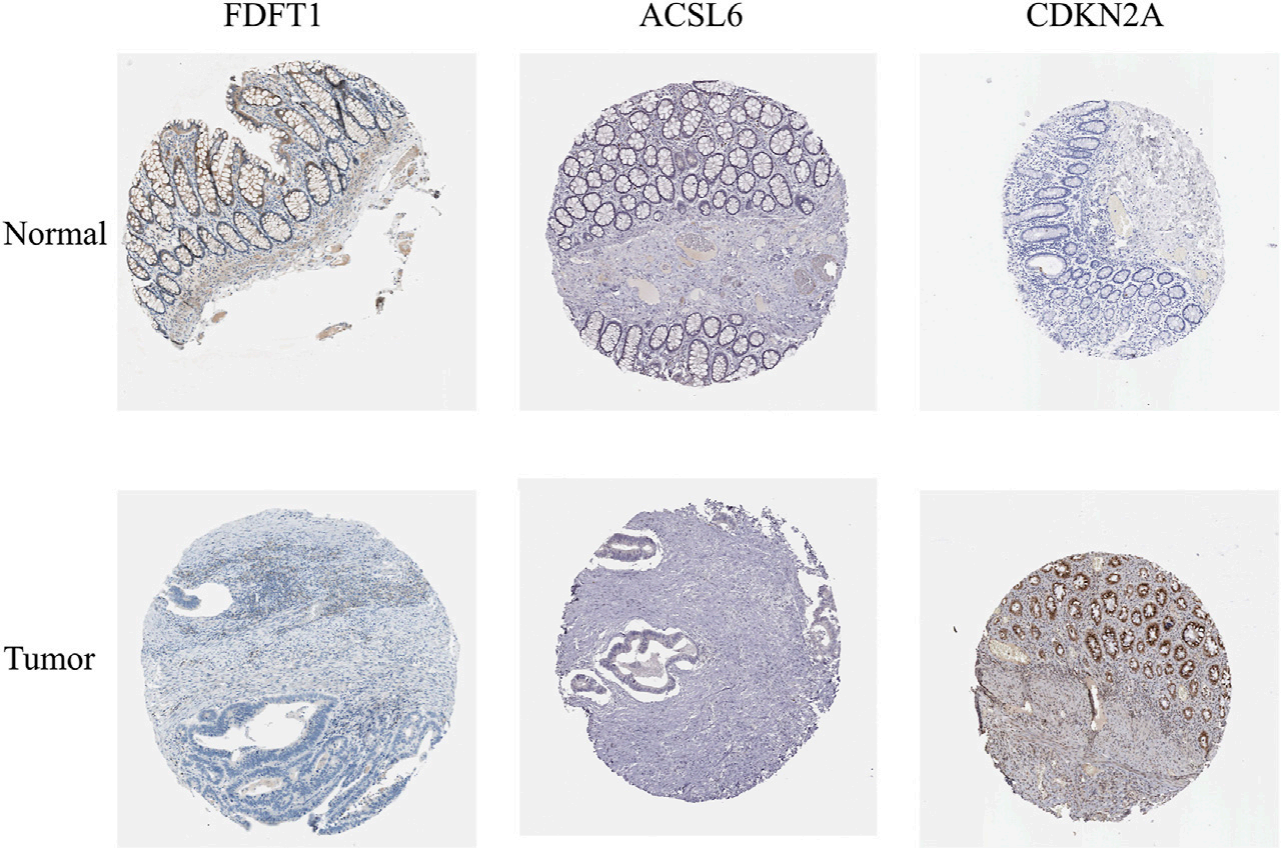
Verification of the expressions of ferroptosis-associated genes in CRC and normal colorectal tissues using HPA database.

**FIGURE 12:**
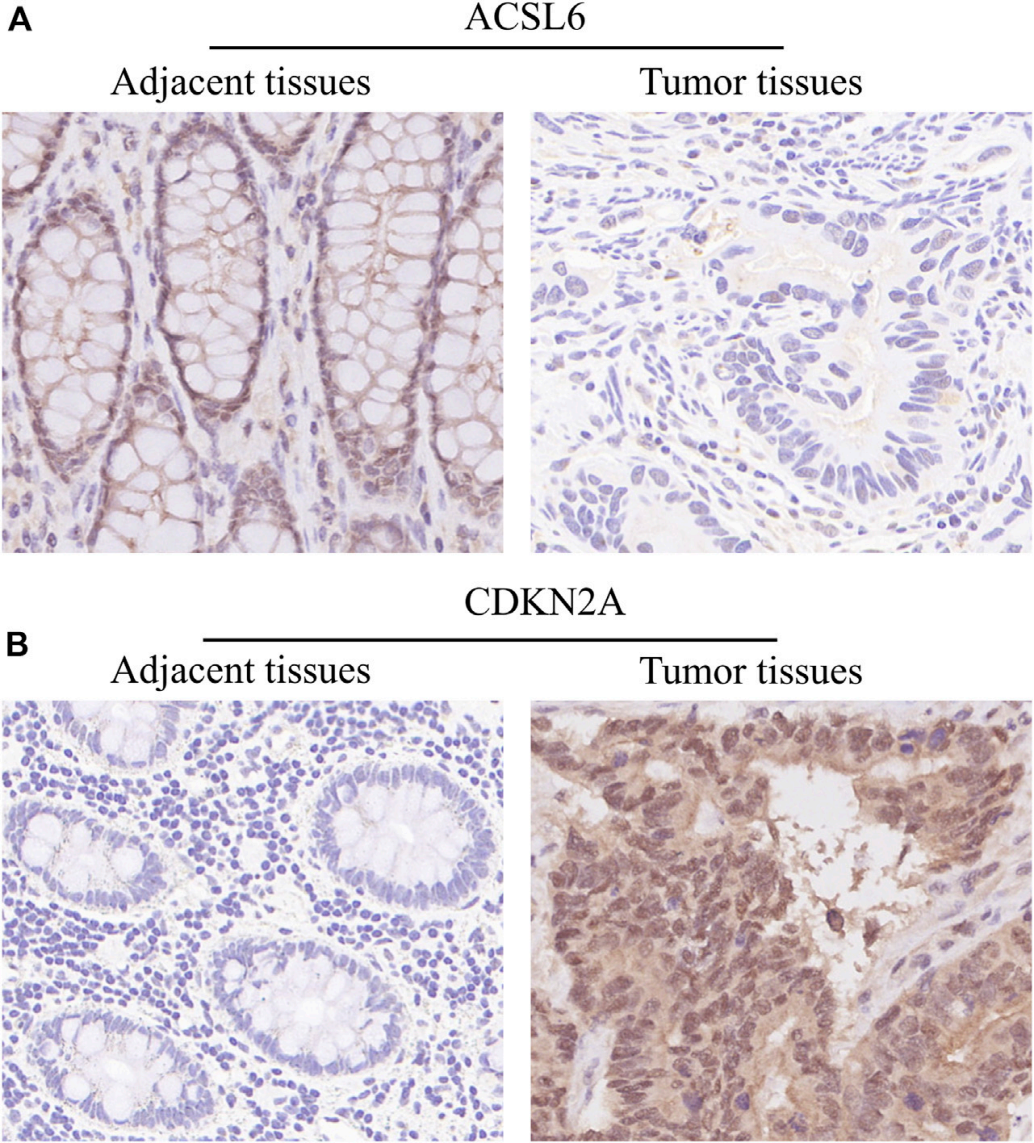
Verify the translational expression of ferroptosis-associated in CRC and normal tissues. **(A)** The representative images of ACSL6 in the adjacent tissues and tumor tissues. **(B)** The representative images of CDKN2A in the adjacent tissues and tumor tissues.

In order to determine the expressions of 3 ferroptosis-associated genes in CRC, the human protein atlas database was used. Immunohistochemical findings were consistent with the transcriptional levels. Expression of CDKN2A was obviously increased in CRC tissue as compared with normal colorectal tissue. However, protein expressions of ACSL6 and FDFT1 were relatively decreased in CRC tissue (Figure 11). We also performed immunohistochemistry against ACSL6 and CDKN2A in the adjacent tissues and CRC tissues. The representative images of immunohistochemistry were shown in Figures 12A,B.

## Discussion

CRC is one of the deadliest carcinomas worldwide, and nearly one-third of patients present with metastasis (Xie et al., 2020). Although CRC patients, particularly those at an advanced stage, achieve significant benefits from new treatments according to individual genomic data, molecular markers and specific tumor location (Jackson and Chester, 2015; Abubakar and Gan, 2016), improving early detection can better reduce the incidence and mortality of CRC. In recent years, multiple gene signature-based risk assessment models have been increasingly used for predicting the prognosis of CRC patients (Yang et al., 2020; Yuan et al., 2021; Yue et al., 2021), which can provide more accurate prediction of prognosis and survival than single gene biomarker. Furthermore, the utilization of mRNA signatures based on tumor and microenvironment features has a better predictive effect on CRC (Zhu et al., 2020; Wang et al., 2021). However, few study has been concerned on the prognosis potential of ferroptosis-associated gene signatures in CRC. In this study, the relationships of 105 ferroptosis-associated genes with OS and the infiltration of immune cells were explored in CRC. A novel prognostic risk signature composed of only 3 ferroptosis-associated genes was built in CRC patients.

Ferroptosis is a new form of cell death, characterized by excessively accumulated iron-dependent lipid hydroperoxide, which was firstly reported in 2012 (Wang et al., 2020). More and more evidences suggest that ferroptosis exerts an important effect on carcinogenesis and treatment of CRC (Hassannia et al., 2019; Liang et al., 2019). Additionally, the expressional level of ferroptosis-associated genes such as GPX4 and SLC7A11 and their susceptibility to ferroptosis are sharply elevated in CRC patients, suggesting that CRC patients may be vulnerable to ferroptosis (Chen et al., 2020). In this study, a new risk signature of ferroptosis was developed, which outperformed other conventional prognostic factors such as age, sex and pathological stage in predicting the survival of CRC patients. The differential expression analysis, univariate and multivariate Cox regression analysis were combinedly used to identify the ferroptosis-associated genes that had been shown to have explicit prognostic value in CRC. Furthermore, our risk model containing just 3 genes was more convenient for clinicians to use in clinic than those models containing multiple genes. This signature was applied to establish a predictive prognostic nomogram model, which would facilitate developing a short-term treatment strategy for CRC patients. The risk signature included 3 ferroptosis-associated genes: FDFT1, ACSL6 and CDKN2A.

To further investigate the effects of above-mentioned three genes on CRC, their mRNA expression levels and major molecular functions were determined and analysed. Based on their hazard ratios, CDKN2A was identified as the risk associated gene, while FDFT1 and ACSL6 as the protective genes. Cyclin-dependent kinase inhibitor 2 (CDKN2A) gene located at chromosome 9p21 is an important cell cycle regulator, which encodes p16INK4a and inhibits CDK4/6 in the cellular cytoplasm (Bihl et al., 2012). Chen et al. reported that CDKN2A can trigger a cell cycle arrest at G1/G2 phase and contribute significantly to cancerigenesis through enhancement of p53-dependent transactivation and ferroptosis (Chen et al., 2017). Moreover, CDKN2A increases the cellular sensitivity to reactive oxygen species (ROS)-induced ferroptosis in a p53 independent fashion, and CDKN2A depletion reduces the risk of ROS-induced cell death (Chen et al., 2017). It has been demonstrated that CDKN2A is silenced in about 30% of CRC (Kohonen-Corish et al., 2014), and hypermethylation of CDKN2A may be correlated with a worse prognosis in CRC patients (Xing et al., 2013). Additionally, it has been reported that CDKN2A is linked to the CpG island methylator phenotype (CIMP) in colon carcinoma, which can promote methylation-mediated gene silencing (Shima et al., 2011). CDKN2A inhibition combined with transcatheter arterial embolization (TAE) treatment can facilitate cancer-cell necrosis in rats with hepatic carcinoma (Gade et al., 2017). It has been revealed that the farnesyl-diphosphate farnesyltransferase 1 (FDFT1) can encode a membrane associated enzyme, which exerts an important effect in cholesterol biosynthesis and ferroptosis (Shimada et al., 2016). In addition, accumulated evidence has revealed that FDFT1 has a key effect on carcinoma, especially in metabolic reprogramming, cell proliferation, and invasion. It has been confirmed by a study that downregulated FDFT1 is related to late tumor progression and worse prognosis in CRC, and FDFT1 suppresses the tumorigenesis through negative regulation of AKT/mTOR/HIF1α signaling pathway (Weng et al., 2020). Moreover, somatic variant analysis indicated that FDFT1 mutation only occurs constantly in the patients with hepatic metastasis, implying that FDFT1-targeted treatment in CRC, particularly in patients with hepatic metastasis, can be a viable strategy (Ma et al., 2019). Acyl-CoA synthetase long-chain family member 6 (ACSL6) is a form present in plasma membrane and displays a high activity with fatty acid (Lopes-Marques et al., 2013). Data analysis revealed that ACSL6 has emerged as a potential tumor suppressor gene in leukemia (Chen et al., 2016). In addition, downregulation of ACSL6 has been found in most carcinoma, except in CRC (Chen et al., 2016). It was detected that miRNAs Let-7c and let-7e targeting against ACSL6 mRNA are decreased in CRC tissue, thus leading to the fact that overexpressed ACSL6 promotes cancerous cells proliferation (Angius et al., 2019). Furthermore, overexpressed ACSL6 in CRC cells can promote fatty acid synthesis, suppress mitochondrial respiration, and increase glycolytic activity, which enhances cell proliferation through providing intermediate metabolites and energy. In general, 3 genes could be classified into 3 categories based on their molecular functions: iron metabolism (CDKN2A), energy metabolism (FDFT1), and lipid metabolism (ACSL6). Although there were few studies on the effects of the above 3 highly connected genes on CRC, we discovered a correlation of decreased FDFT1 and ACSL6 and increased CDKN2A with a worse OS in CRC patients in this study, and more studies are needed to investigate its underlying mechanism.

Immunotherapy, which plays a vital role in antitumor treatment, has been paid more and more attention. Recent studies indicated that ferroptosis may be critical in tumor immunotherapy (Lang et al., 2019; Stockwell and Jiang, 2019; Wang et al., 2019; Zeng et al., 2021). Under the presence of ferroptosis, the antitumor immunity can be regulated by the cells *via* releasing chemotaxis and interacting with immune cells such as CD8^+^ T cells and NK cells (Wang et al., 2019; Zitvogel and Kroemer, 2019; Chen et al., 2021). In this study, the fraction of resting NK cells, memory-resting CD4^+^ T cells were decreased, M0 and M1 macrophages were increased in high-risk group, indicating that ferroptosis has an obvious immune suppressive feature, which leads to a worse OS in CRC patients in high-risk group. Immune checkpoints such as PD1, PD-L1, CTLA-4, and LAG3 are important in tumorigenesis, which can promote tumor immunosuppressive effects. Whereas, tumor cells can defend themselves against attack by stimulating immune checkpoint targets. Therefore, immune checkpoint blockade therapy is a crucial for progression of antitumor immunotherapies, which activates the natural cancer-selective killing activity of T cells. According to recent studies, ferroptosis can regulate the anticancer activity of CD8^+^ T cells *via* increasing tumor cell sensitivity to PD-1/PD-L1 or CTLA-4 blocking therapy (Lang et al., 2019; Wang et al., 2019). Consistent with the above findings, immune checkpoints such as PD1, PD-L1 and CTLA-4 were increased in the high-risk group in this study. The above result suggests that patients in the high-risk group can be susceptible to immunotherapy or checkpoint inhibitor-based immunotherapy. Combined application of immunotherapy and ferroptosis-targeting therapy would be a viable treatment strategy.

The TMB is thought to be associated with the number of neoantigens in tumors and is important in predicting the efficacy of immune checkpoint inhibitors (Allgauer et al., 2018). Recent studies have shown that TMB is an important biomarker for predicting the response to PD-L1 therapy (Topalian et al., 2016). Our study showed that the TMB in the high-risk group exceeded that in the low-risk group, which proved that immune checkpoint inhibitors might be more effective in the high-risk group.

In summary, we develop a risk signature for CRC according to ferroptosis-associated genes, which has a good prognosis effect and reflects the link between immune microenvironment and the outcome of CRC patients. Importantly, this study provides novel insights into the molecular mechanism underlying ferroptosis in CRC patients, and also indicates that ferroptosis-targeted treatment will be a promising treatment for CRC patients. Nevertheless, this study has a few limitations. First of all, the data in this retrospective study were retrieved from public datasets, so a prospective study with more large-scale data need to be performed to testify its clinical utility of present findings. Secondly, the clinical data provided in public datasets is limited, which may decrease the reliability of nomogram we built in this study. Finally, the risk signature in this study was defined by bioinformatics analysis, further functional experiments are needed to verify the present findings and investigate their underlying mechanisms before clinical application.

## Data Availability

RNA-seq data and clinical information applied to support the findings of this study were downloaded from the Cancer Genome Atlas (TCGA) (https://cancergenome.nih.gov/) andGene Expression Omnibus (GEO) repository (GSE39582).
