# Supplementary figures and images for: A ferroptosis associated gene signature for predicting prognosis and immune responses in patients with colorectal carcinoma
Source: Front Genet. 2022 Sep 8;13:971364. doi: 10.3389/fgene.2022.971364 (PMC9493326; doi:10.3389/fgene.2022.971364)

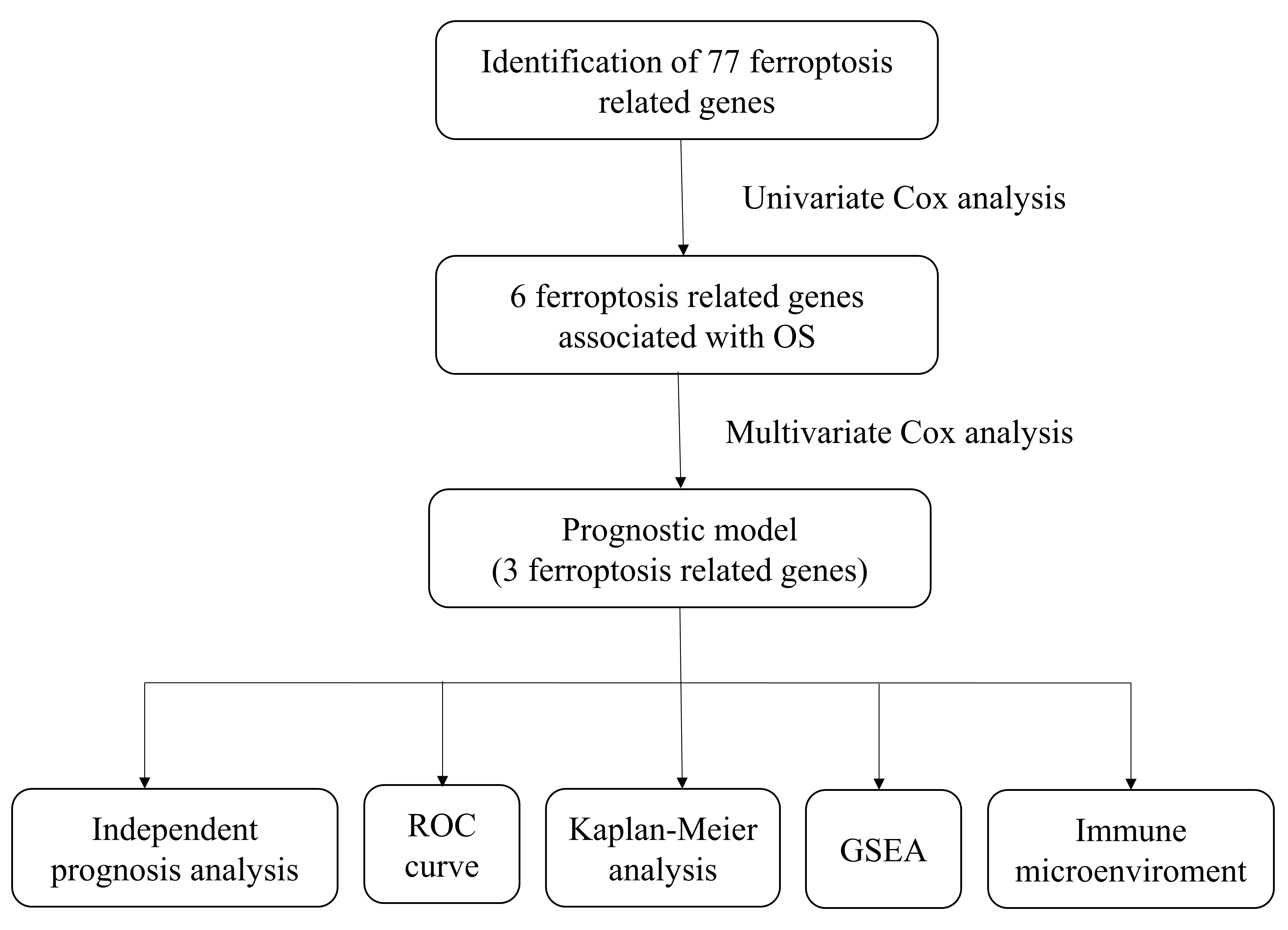

Supplement: Supplementary file 1 [file Image1.TIFF]
