# Supplementary material for: A ferroptosis associated gene signature for predicting prognosis and immune responses in patients with colorectal carcinoma
Source: Front Genet. 2022 Sep 8;13:971364. doi: 10.3389/fgene.2022.971364 (PMC9493326; doi:10.3389/fgene.2022.971364)
Supplement: Supplementary file 2 [file Table1.DOC]

Table S2. 105 Ferroptosis Related Genes Downloaded from the GeneCards.

| **Gene Symbol** | **Description** | **Category** |
| --- | --- | --- |
| GPX4 | Glutathione Peroxidase 4 | Protein Coding |
| AIFM2 | Apoptosis Inducing Factor Mitochondria Associated 2 | Protein Coding |
| TP53 | Tumor Protein P53 | Protein Coding |
| ACSL4 | Acyl-CoA Synthetase Long Chain Family Member 4 | Protein Coding |
| SLC7A11 | Solute Carrier Family 7 Member 11 | Protein Coding |
| VDAC2 | Voltage Dependent Anion Channel 2 | Protein Coding |
| VDAC3 | Voltage Dependent Anion Channel 3 | Protein Coding |
| ATG5 | Autophagy Related 5 | Protein Coding |
| ATG7 | Autophagy Related 7 | Protein Coding |
| NCOA4 | Nuclear Receptor Coactivator 4 | Protein Coding |
| HMOX1 | Heme Oxygenase 1 | Protein Coding |
| SLC3A2 | Solute Carrier Family 3 Member 2 | Protein Coding |
| ALOX15 | Arachidonate 15-Lipoxygenase | Protein Coding |
| BECN1 | Beclin 1 | Protein Coding |
| PRKAA1 | Protein Kinase AMP-Activated Catalytic Subunit Alpha 1 | Protein Coding |
| SAT1 | Spermidine/Spermine N1-Acetyltransferase 1 | Protein Coding |
| NF2 | Neurofibromin 2 | Protein Coding |
| YAP1 | Yes1 Associated Transcriptional Regulator | Protein Coding |
| FTH1 | Ferritin Heavy Chain 1 | Protein Coding |
| TF | Transferrin | Protein Coding |
| TFRC | Transferrin Receptor | Protein Coding |
| FTL | Ferritin Light Chain | Protein Coding |
| CYBB | Cytochrome B-245 Beta Chain | Protein Coding |
| GSS | Glutathione Synthetase | Protein Coding |
| CP | Ceruloplasmin | Protein Coding |
| PRNP | Prion Protein | Protein Coding |
| SLC11A2 | Solute Carrier Family 11 Member 2 | Protein Coding |
| SLC40A1 | Solute Carrier Family 40 Member 1 | Protein Coding |
| STEAP3 | STEAP3 Metalloreductase | Protein Coding |
| ACSL1 | Acyl-CoA Synthetase Long Chain Family Member 1 | Protein Coding |
| GCLC | Glutamate-Cysteine Ligase Catalytic Subunit | Protein Coding |
| MAP1LC3A | Microtubule Associated Protein 1 Light Chain 3 Alpha | Protein Coding |
| MAP1LC3B | Microtubule Associated Protein 1 Light Chain 3 Beta | Protein Coding |
| SLC39A14 | Solute Carrier Family 39 Member 14 | Protein Coding |
| SLC39A8 | Solute Carrier Family 39 Member 8 | Protein Coding |
| ACSL5 | Acyl-CoA Synthetase Long Chain Family Member 5 | Protein Coding |
| GCLM | Glutamate-Cysteine Ligase Modifier Subunit | Protein Coding |
| PCBP1 | Poly(RC) Binding Protein 1 | Protein Coding |
| PCBP2 | Poly(RC) Binding Protein 2 | Protein Coding |
| ACSL3 | Acyl-CoA Synthetase Long Chain Family Member 3 | Protein Coding |
| ACSL6 | Acyl-CoA Synthetase Long Chain Family Member 6 | Protein Coding |
| SAT2 | Spermidine/Spermine N1-Acetyltransferase Family Member 2 | Protein Coding |
| FTMT | Ferritin Mitochondrial | Protein Coding |
| LPCAT3 | Lysophosphatidylcholine Acyltransferase 3 | Protein Coding |
| MAP1LC3C | Microtubule Associated Protein 1 Light Chain 3 Gamma | Protein Coding |
| MAP1LC3B2 | Microtubule Associated Protein 1 Light Chain 3 Beta 2 | Protein Coding |
| BAP1 | BRCA1 Associated Protein 1 | Protein Coding |

| PRDX6 | Peroxiredoxin 6 | Protein Coding |
| --- | --- | --- |
| SESN2 | Sestrin 2 | Protein Coding |
| ARNTL | Aryl Hydrocarbon Receptor Nuclear Translocator Like | Protein Coding |
| CISD1 | CDGSH Iron Sulfur Domain 1 | Protein Coding |
| PROM2 | Prominin 2 | Protein Coding |
| NEDD4 | NEDD4 E3 Ubiquitin Protein Ligase | Protein Coding |
| CA9 | Carbonic Anhydrase 9 | Protein Coding |
| ELAVL1 | ELAV Like RNA Binding Protein 1 | Protein Coding |
| NFE2L2 | Nuclear Factor, Erythroid 2 Like 2 | Protein Coding |
| ITGA6 | Integrin Subunit Alpha 6 | Protein Coding |
| PRKAA2 | Protein Kinase AMP-Activated Catalytic Subunit Alpha 2 | Protein Coding |
| FANCD2 | FA Complementation Group D2 | Protein Coding |
| LAMP2 | Lysosomal Associated Membrane Protein 2 | Protein Coding |
| ALOX12 | Arachidonate 12-Lipoxygenase, 12S Type | Protein Coding |
| CD44 | CD44 Molecule (Indian Blood Group) | Protein Coding |
| MAPK1 | Mitogen-Activated Protein Kinase 1 | Protein Coding |
| MYC | MYC Proto-Oncogene, BHLH Transcription Factor | Protein Coding |
| EGLN1 | Egl-9 Family Hypoxia Inducible Factor 1 | Protein Coding |
| GOT1 | Glutamic-Oxaloacetic Transaminase 1 | Protein Coding |
| MAP3K5 | Mitogen-Activated Protein Kinase Kinase Kinase 5 | Protein Coding |
| ATF4 | Activating Transcription Factor 4 | Protein Coding |
| FH | Fumarate Hydratase | Protein Coding |
| HELLS | Helicase, Lymphoid Specific | Protein Coding |
| SOCS1 | Suppressor Of Cytokine Signaling 1 | Protein Coding |
| OTUB1 | OTU Deubiquitinase, Ubiquitin Aldehyde Binding 1 | Protein Coding |
| CARS1 | Cysteinyl-TRNA Synthetase 1 | Protein Coding |
| MIR9-1 | MicroRNA 9-1 | RNA Gene |
| MIR137 | MicroRNA 137 | RNA Gene |
| HMGB1 | High Mobility Group Box 1 | Protein Coding |
| LINC00336 | Long Intergenic Non-Protein Coding RNA 336 | RNA Gene |
| NFS1 | NFS1 Cysteine Desulfurase | Protein Coding |
| PEBP1 | Phosphatidylethanolamine Binding Protein 1 | Protein Coding |
| RB1 | RB Transcriptional Corepressor 1 | Protein Coding |
| G3BP1 | G3BP Stress Granule Assembly Factor 1 | Protein Coding |
| LINC00472 | Long Intergenic Non-Protein Coding RNA 472 | RNA Gene |
| EPAS1 | Endothelial PAS Domain Protein 1 | Protein Coding |
| HILPDA | Hypoxia Inducible Lipid Droplet Associated | Protein Coding |
| PRC1 | Protein Regulator Of Cytokinesis 1 | Protein Coding |
| NGB | Neuroglobin | Protein Coding |
| MDM2 | MDM2 Proto-Oncogene | Protein Coding |
| TIGAR | TP53 Induced Glycolysis Regulatory Phosphatase | Protein Coding |
| VDAC1 | Voltage Dependent Anion Channel 1 | Protein Coding |
| HSPB1 | Heat Shock Protein Family B (Small) Member 1 | Protein Coding |
| HSPA5 | Heat Shock Protein Family A (Hsp70) Member 5 | Protein Coding |
| CDKN2A | Cyclin Dependent Kinase Inhibitor 2A | Protein Coding |
| CASP8 | Caspase 8 | Protein Coding |
| CFTR | CF Transmembrane Conductance Regulator | Protein Coding |
| AURKA | Aurora Kinase A | Protein Coding |
| MIF | Macrophage Migration Inhibitory Factor | Protein Coding |

| RIPK1 | Receptor Interacting Serine/Threonine Kinase 1 | Protein Coding |
| --- | --- | --- |
| MUC1 | Mucin 1, Cell Surface Associated | Protein Coding |
| ALOX15B | Arachidonate 15-Lipoxygenase Type B | Protein Coding |
| ANO6 | Anoctamin 6 | Protein Coding |
| GUCY1A1 | Guanylate Cyclase 1 Soluble Subunit Alpha 1 | Protein Coding |
| MT1G | Metallothionein 1G | Protein Coding |
| MIR7-1  FDFT1  HMGCR | MicroRNA 7-1  Farnesyl-Diphosphate Farnesyltransferase 1  3-Hydroxy-3-Methylglutaryl-CoA Reductase | RNA Gene  Protein Coding  Protein Coding |

GeneCards: https://[www.genecards.org/](http://www.genecards.org/)
