# Supplementary material for: A ferroptosis associated gene signature for predicting prognosis and immune responses in patients with colorectal carcinoma
Source: Front Genet. 2022 Sep 8;13:971364. doi: 10.3389/fgene.2022.971364 (PMC9493326; doi:10.3389/fgene.2022.971364)
Supplement: Supplementary file 3 [file Table2.DOC]

**Table S1 Clinical Characteristics of CRC patients in the TCGA and GEO cohorts**

| Characteristics | Subgroup | TCGA(n=453) | GEO(n=579) |
| --- | --- | --- | --- |
| Age | ＜50 | 54 | 67 |
|  | ≥50 | 399 | 512 |
| Gender | Female | 214 | 260 |
|  | Male | 239 | 319 |
| Survival status | Dead | 100 | 194 |
|  | Living | 353 | 385 |
| Stage | Stage I | 75 | 37 |
|  | Stage II | 175 | 269 |
|  | Stage III | 128 | 209 |
|  | Stage IV | 64 | 60 |
|  | Unknown | 11 | 4 |
| T | T1 | 11 | 12 |
|  | T2 | 77 | 48 |
|  | T3 | 308 | 376 |
|  | T4 | 56 | 119 |
|  | Tis  Unknown | 1  0 | 3  21 |
| N | N0 | 266 | 311 |
|  | N1 | 105 | 136 |
|  | N2  N3  Unknown | 82  0  0 | 100  6  26 |
| M | M0 | 332 | 496 |
|  | M1  Mx | 64  50 | 61  2 |
|  | unknown | 7 | 20 |
